# Expression Profiles of Exosomal MicroRNAs Derived from Cerebrospinal Fluid in Patients with Congenital Hydrocephalus Determined by MicroRNA Sequencing

**DOI:** 10.1155/2022/5344508

**Published:** 2022-03-04

**Authors:** Shiyu Chen, Hao Li, Jicui Zheng, Lili Hao, Tianrui Jing, Peixuan Wu, Bowen Zhang, Duan Ma, Jing Zhang, Jing Ma

**Affiliations:** ^1^Institutes of Biomedical Sciences, Fudan University, Shanghai, China; ^2^Children's Hospital of Fudan University, Shanghai, China; ^3^School of Basic Medical Sciences, Fudan University, Shanghai, China; ^4^ENT Institute, Department of Facial Plastic and Reconstructive Surgery, Eye & ENT Hospital, Fudan University, Shanghai, China

## Abstract

**Purpose:**

Congenital hydrocephalus is one of the most common birth defects worldwide. Exosomal microRNAs (miRNAs) in body fluids have been implicated in many diseases. However, their involvement in cerebrospinal fluid from congenital hydrocephalus is not well understood. This study is aimed at investigating the role of dysregulated exosomal miRNAs in congenital hydrocephalus.

**Methods:**

We collected cerebrospinal fluid samples from 15 congenital hydrocephalus patients and 21 control subjects. We used miRNA sequencing to generate exosomal miRNA expression profiles in three pairs of samples. We identified 31 differentially expressed exosomal miRNAs in congenital hydrocephalus and predicted their target mRNAs.

**Results:**

Three microRNAs (hsa-miR-130b-3p, hsa-miR-501-5p, and hsa-miR-2113) were selected according to their fold changes and the function of their target mRNAs, and only hsa-miR-130b-3p and hsa-miR-501-5p were confirmed their expression levels in all samples. Moreover, upregulated hsa-miR-130b-3p might mediate the downregulation of the phosphatase and tensin homolog gene (PTEN), which has been associated with hydrocephalus, via binding to its 3′-untranslated region by dual-luciferase reporter assay.

**Conclusion:**

This study implicates that abnormally expressed exosomal miRNAs in cerebrospinal fluid may be involved in the pathomechanism of congenital hydrocephalus.

## 1. Introduction

Congenital hydrocephalus (CH) is among the top five birth defects worldwide, with a prevalence of 4.65 in 10,000 births [1]. As one of the most common congenital central nervous system anomalies, CH results from the accumulation of cerebrospinal fluid in the brain ventricles, leading to severe neurological damage. The main clinical manifestations are ventriculomegaly, increased intracranial pressure, and brain dysfunction, which may lead to dysgnosia, while ventriculomegaly can critically impair the developmental processes affecting various anatomical and functional aspects of brain maturation [2]. Ventriculoperitoneal shunts are common treatments for CH; however, intracranial pressure may be normal or even low in some patients, including those with other structural brain abnormalities, and surgical shunting may fail to improve the neurological impairment in those patients [2, 3]. Furthermore, there is a high risk of postoperative complications, such as intracranial infection. It is therefore important to explore the etiology and pathogenesis of CH to improve its prevention and treatment.

The underlying causes of CH are currently not well understood, but it is generally believed to be a multifactorial disease involving genetic and environmental interactions. CH may be associated with chromosomal (e.g., abnormality of chromosome 6q and Xp22.33) or single gene abnormalities [1], and mutations of at least 43 genes/loci have been associated with CH in animal models or patients [4]. In addition, gene expression disorders may also be involved in the etiology of CH [5]. Among the factors regulating gene expression, microRNAs (miRNAs; miR) are responsible for modulating nearly one-third of genes and thus regulating a variety of physiological processes [6]. Although miRNAs are found widely both inside and outside cells, about 70% of all miRNAs are stably distributed in exosomes, instead of in their host cells [7]. Exosomes are phospholipid bilayer vesicles, with a diameter of 40–100 nm. They are secreted by cells and form a cell-to-cell information transmission system by carrying various biological molecules (protein, DNA, mRNA, and miRNA), which in turn affect the physiological state of the cells and are closely related to the occurrence and process of a variety of diseases [8]. Exosomes can cross the blood-brain barrier and are readily accessible in various human biofluids, including cerebrospinal fluid [9, 10]. The characteristics of exosomes with a substantial miRNA content ensure to act as promising biomarkers in some diseases. For instance, exosomal miR-181-5p from blood samples was identified as a potential diagnostic biomarker for early-stage non-small-cell lung cancer [8], and exosomal miR-150 and miR-21 from blood samples were indicated as biomarkers for the early detection of colorectal cancer [11]. These exosomal miRNAs can be delivered to recipient cells to exert their functions [12, 13] by affecting the translation or stability of their target mRNAs via direct interactions [9]. The existence and importance of exosomal miRNAs in cerebrospinal fluid have been confirmed in several neurological disorders, such as Alzheimer's disease [14], Parkinson's disease [15], and intraventricular hemorrhage (IVH) in preterm infants [16]. However, the role of exosomal miRNAs in the cerebrospinal fluid in CH remains unknown.

In the current study, we characterized the exosomal miRNA profile of cerebrospinal fluid from patients with CH using miRNA sequencing and bioinformatics analysis and focused on three miRNAs (miR-130b-3p, miR-501-5p, and miR-2113). We verified the differential expression of the above three miRNAs in patients with CH, and we also investigated the expression of phosphatase and tensin homolog gene (PTEN), which was associated with hydrocephalus in previous reports [17] via the mechanism of these miRNAs.

## 2. Materials and Methods

### 2.1. Patients and Samples

Cerebrospinal fluid samples were obtained from the age-sex-matched 15 CH patients and 21 control subjects (CS) from the Children's Hospital of Fudan University. The diagnosis of CH was carried out at the Children's Hospital of Fudan University and excluded trauma, tumor, secondary hydrocephalus, and infection. The CS were the patients diagnosed with three types of secondary hydrocephalus (intracranial space-occupying lesions, intracranial hemorrhage, and congenital tethered cord syndrome). The cerebrospinal fluid samples were maintained in RNA-free centrifuge tubes after surgery and stored at −80°C. miRNA sequencing was carried out using cerebrospinal fluid samples from three CH patients and three CS (Table 1). Cerebrospinal fluid samples from 15 CH patients and 21 CS were used for further real-time quantitative qPCR analysis (Tables 2 and 3).

### 2.2. Exosome Isolation and RNA Extraction

The cerebrospinal fluid samples were centrifuged for 30 min at 900*g* and 4°C to remove cells and large debris. The supernatant was collected and filtered through a 0.22 *μ*m filter to remove additional cellular fragments. Exosomes were isolated and purified from the supernatant using an exoEasy Maxi Kit (Qiagen, Hilden, Germany) following the manufacturer's protocol. Total exosomal RNA was extracted using TRIzol reagent (Invitrogen, USA) according to the manufacturer's instructions. The RNA quality and concentration were evaluated based on the optical density 260/280 and 260/230 ratios using a Nano Drop ND-2000 spectrophotometer (Thermo Fisher Scientific, Waltham, MA, USA). RNA integrity was assessed by agarose gel electrophoresis.

### 2.3. miRNA Sequencing and Data Analysis

Exosomal miRNA libraries were constructed using total RNA samples from exosomes and assessed using an Agilent 2100 Bioanalyzer (Agilent, California, USA). The 3′ and 5′ adapters were ligated to total RNA samples, respectively, and cDNAs were synthesized with the adapter-ligated miRNAs and used as templates for polymerase chain reaction (PCR) amplification. Amplified fragments of 135-155 base pairs were selected to construct miRNA libraries, which were then denatured with 0.1 M NaOH and sequenced using a TruSeq Rapid SR Cluster Kit (Illumina, CA, USA) with Illumina NextSeq 500, according to the manufacturer's instructions. Raw reads were subjected to quality control to assess the suitability of the raw data for subsequent analysis. Trimmed data were obtained by removing the 3′ adapters and shorter reads (≤15 base pairs) from the raw data. The subsequent reads were aligned with the human reference genome annotated with miRNA to generate an miRNA expression value (counts per million reads; CPM) using miRDeep2 [17]. The detected miRNAs were determined based on an average CPM value > 1.

### 2.4. Differential Expression of Exosomal miRNAs

Differentially expressed exosomal miRNAs were identified using edgeR with a threshold fold change (FC) > 1.5 and *P* value ≤ 0.05. We used the CPM value of significantly expressed exosomal miRNAs to perform hierarchical clustering analysis and reveal the expression patterns of the exosomal miRNAs and samples. Scatter plots were generated to assess the distribution trends of the miRNAs in the CS and CH patients. Differentially expressed exosomal miRNAs were screened based on a log_2_FC and −log_10_*P* value to generate volcano plots demonstrating the relationship between the FC of differential expression and statistical significance.

### 2.5. The Target Genes of miRNA Prediction, Functional Annotation, and Pathway Enrichment

The miRDB and TargetScan algorithms were used to predict the target genes of exosomal miRNAs that were differentially expressed between the CS and CH patients. Functional enrichment of the target genes was then determined by Gene Ontology (GO) (http://www.geneontology.org/) and Kyoto Encyclopedia of Genes and Genomes (KEGG) (http://www.genome.jp/kegg) analyses.

### 2.6. Quantitative Polymerase Chain Reaction (qPCR)

cDNAs were synthesized from 1 *μ*g total RNA using a PrimeScript RT Reagent Kit with gDNA Eraser (Takara, Tokyo, Japan), and qPCR was conducted with SYBR Premix Ex Taq™ (Takara) on a StepOnePlus™ Real-Time PCR System (Thermo Fisher Scientific). The relative expression levels of miRNAs and mRNAs were normalized to the housekeeping gene U6 and glyceraldehyde 3-phosphate dehydrogenase (GAPDH), respectively, and were calculated by the relative quantification method (2^−*ΔΔ*Ct^).

The primers used were as follows: hsa-miR-2113-GSP: GGGGATTTGTGCTTGGCTC, hsa-miR-2113-R: GTGCGTGTCGTGGAGTCG; hsa-miR-130b-3p-GSP: GGGCAGTGCAATGATGAAA, hsa-miR-130b-3p-R: GTGCGTGTCGTGGAGTCG; hsa-miR-501-5p-GSP: GGAGAATCCTTTGTCCCTGG, hsa-miR-501-5p-R: GTGCGTGTCGTGGAGTCG; U6-F: GCTTCGGCAGCACATATACTAAAAT, U6-R: CGCTTCACGAATTTGCGTGTCAT; PTEN-F: ACACGACGGGAAGACAAGTT, PTEN-R: CTGGTCCTGGTATGAAGAATG; and GAPDH-F: GGGAAACTGTGGCGTGAT, GAPDH-R: GAGTGGGTGTCGCTGTTGA.

### 2.7. Cell Culture

Human embryonic kidney 293 (HEK293T) cells were seeded in Dulbecco's modified Eagle's medium (Biological Industries, Kibbutz Beit HaEmek) with 10% foetal bovine serum (Biological Industries) at 37°C in 5% CO_2_. All cell culture dishes and culture plates were purchased from Hangzhou Xinyou Biotechnology Co., Ltd.

### 2.8. Dual-Luciferase Reporter Assay

The recombinant plasmid pGL3-promoter-PTEN-WT (wild-type PTEN 3′-untranslated region (UTR)) and pGL3-promoter-PTEN-Del (deleted PTEN 3′-untranslated region (UTR)) were constructed. Mimics and NC oligonucleotides for hsa-miR-130b-3p were obtained from RiboBio Co., Ltd. (China). HEK293T cells (Cell Bank, Shanghai, China) were seeded in 96-well plates at 1 × 10^4^ cells per well and incubated overnight at 37°C. The respective mimics and NC oligonucleotides were cotransfected into HEK293T cells with pGL3-promoter-PTEN-WT/pGL3-promoter-PTEN-Del and pGL3-Renilla using Lipofectamine 3000 (Invitrogen). Cells were then harvested 48 h after transfection. Both firefly and Renilla luciferase activities were measured using a Dual-Luciferase Reporter Assay System (Promega, USA), and the firefly luciferase activities were normalized to Renilla luciferase activities.

The primers used were as follows: PTEN-3utr-Xba1-F: GCTCTAGAGCtggcaataggacattgtgtc and PTEN-3utr-Xba1-R: GCTCTAGAGCgctgccttttctagcaccaatatgc.

### 2.9. Statistical Analysis

All experiments were repeated three times. All statistical analyses were performed by paired two-tailed Student's *t*-tests using GraphPad Software (GraphPad Inc., La Jolla, CA, USA). A value of *P* < 0.05 was considered significant.

## 3. Results

### 3.1. Analysis of Differentially Expressed Exosomal miRNAs in CH Patients and CS

Differential expression of miRNAs in cerebrospinal fluid exosomes from three CH patients and three CS was analyzed using edgeR. The criteria for differential miRNA expression were an FC threshold of 1.5, *P* value ≤ 0.05, and mean CPM ≥ 1. Log_2_FC was calculated to represent differential miRNA expression, with a positive value indicating upregulation and a negative value indicating downregulation. We identified thousands of differentially expressed human miRNAs.

**Figure 1:**
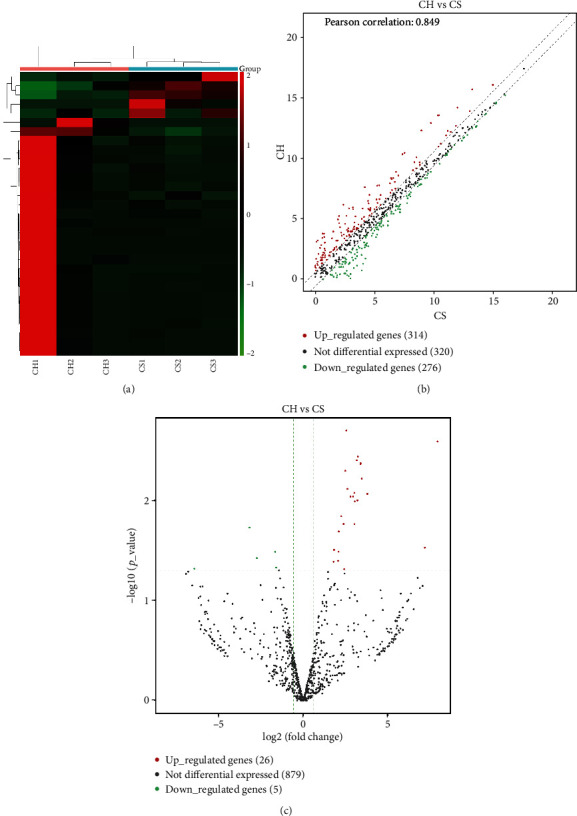
Differential expression profiles of exosomal miRNAs in CH patients and CS. (a) Hierarchical clustering analysis of exosomal miRNAs between the CH patients (CH1, CH2, and CH3) and CS (CS1, CS2, and CS3). Expression values are represented by red and green shades, indicating expressions above and below the median expression level across all samples, respectively. (b) The scatter plot of 910 exosomal miRNAs. Pearson's correlation coefficient was 0.849. The red dots indicate upregulated genes, the green dots indicate downregulated genes, and the black dots indicate nondifferentially expressed genes. (c) The volcano plot of 910 exosomal miRNAs. The fold change threshold is 1.5 and *P* value ≤ 0.05. The red dots indicate upregulated genes, the green dots indicate downregulated genes, and the black dots indicate nondifferentially expressed genes.

We performed differential expression analyses of three miRNAs by hierarchical clustering, scatter plots, and volcano plots, respectively. Hierarchical clustering analysis of exosomal miRNA signal intensities revealed evidence of significant differential expression of exosomal miRNAs between the CH patients and CS (Figure 1(a)). There were 910 miRNAs in the scatter plot, of which 314 and 274 were upregulated and downregulated, respectively (Figure 1(b)). Pearson's correlation coefficient was 0.849. Among these miRNAs, 26 and 5 were significantly upregulated and downregulated in the volcano plot, respectively (Figure 1(c), Table 4).

### 3.2. Target Gene Prediction for Differentially Expressed Exosomal miRNAs

The top ten most upregulated exosomal miRNAs (hsa-miR-129-5p, hsa-miR-130b-3p, hsa-miR-2113, hsa-miR-302d-3p, hsa-miR-320b, hsa-miR-320c, hsa-miR-320d, hsa-miR-320e, hsa-miR-4429, and hsa-miR-137-5p) and the top four most downregulated exosomal miRNAs (hsa-let-7e-3p, hsa-miR-223-5p, hsa-miR-501-5p, and hsa-miR-584-5p) were selected for evaluation. Target genes were predicted using TargetScan and miRDB, generating 4640 potential target genes, including 3542 genes for upregulated and 1098 genes for downregulated miRNAs.

### 3.3. Functional Analysis of Differentially Expressed Exosomal miRNA Target Genes

**Figure 2:**
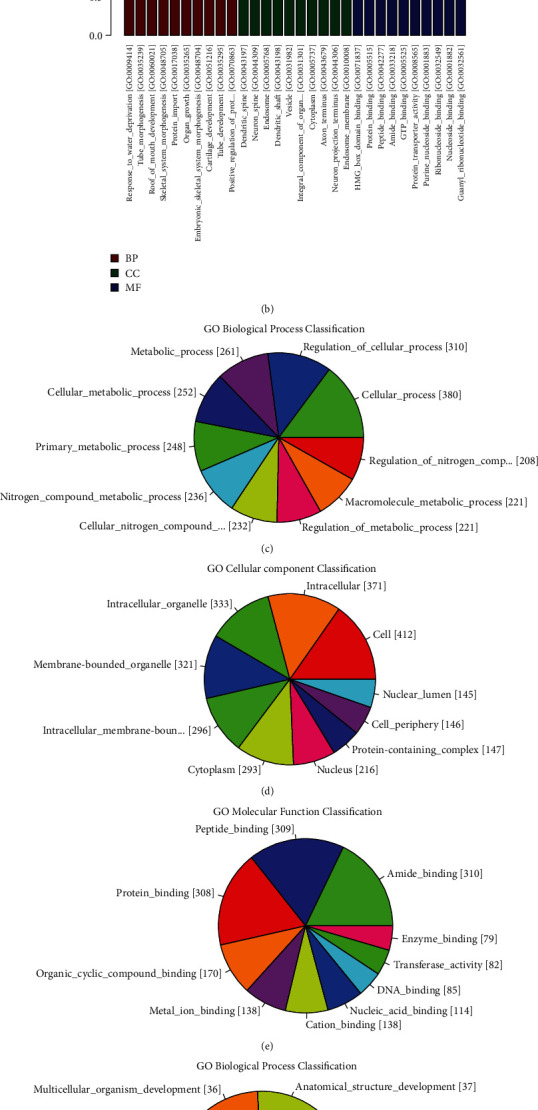
GO terms analysis of the predicted target genes of differentially expressed exosomal miRNAs in CH patients. (a) Enriched GO terms of differentially upregulated miRNA target genes in CH patients compared with CS. (b) Enriched GO terms of differentially downregulated miRNA target genes in CH patients compared with CS. (c, d, e) The top 10 GO terms of biological process, cellular component, and molecular function in differentially upregulated miRNA target genes according to the gene counts included in each term. (f, g, h) The top 10 GO terms of biological process, cellular component, and molecular function in differentially downregulated miRNA target genes according to the gene counts included in each term. BP: biological process; CC: cellular component; MF: molecular function.

To further highlight the functional features of exosomal miRNAs, the target genes were annotated using GO terms. The target genes of upregulated miRNAs were mainly enriched in “regulation of nitrogen compound metabolic process” (208 genes, *P* = 3.14*E* − 12), “nuclear lumen”(145 genes, *P* = 6.31*E* − 10), “double-stranded DNA binding” (47 genes, *P* = 4.34*E* − 07), and so on (Figures 2(a), 2(c), 2(d), and 2(e), Table S1). The target genes of downregulated miRNAs were mainly enriched in “response to water deprivation” (two genes, *P* = 6.25*E* − 04), “dendritic_spine” (four genes, *P* = 7.63*E* − 03), “HMG_box_domain_binding” (two genes, *P* = 3.18*E* − 03), and so on (Figures 2(b), 2(f), 2(g), and 2(h), Table S2).

**Figure 3:**
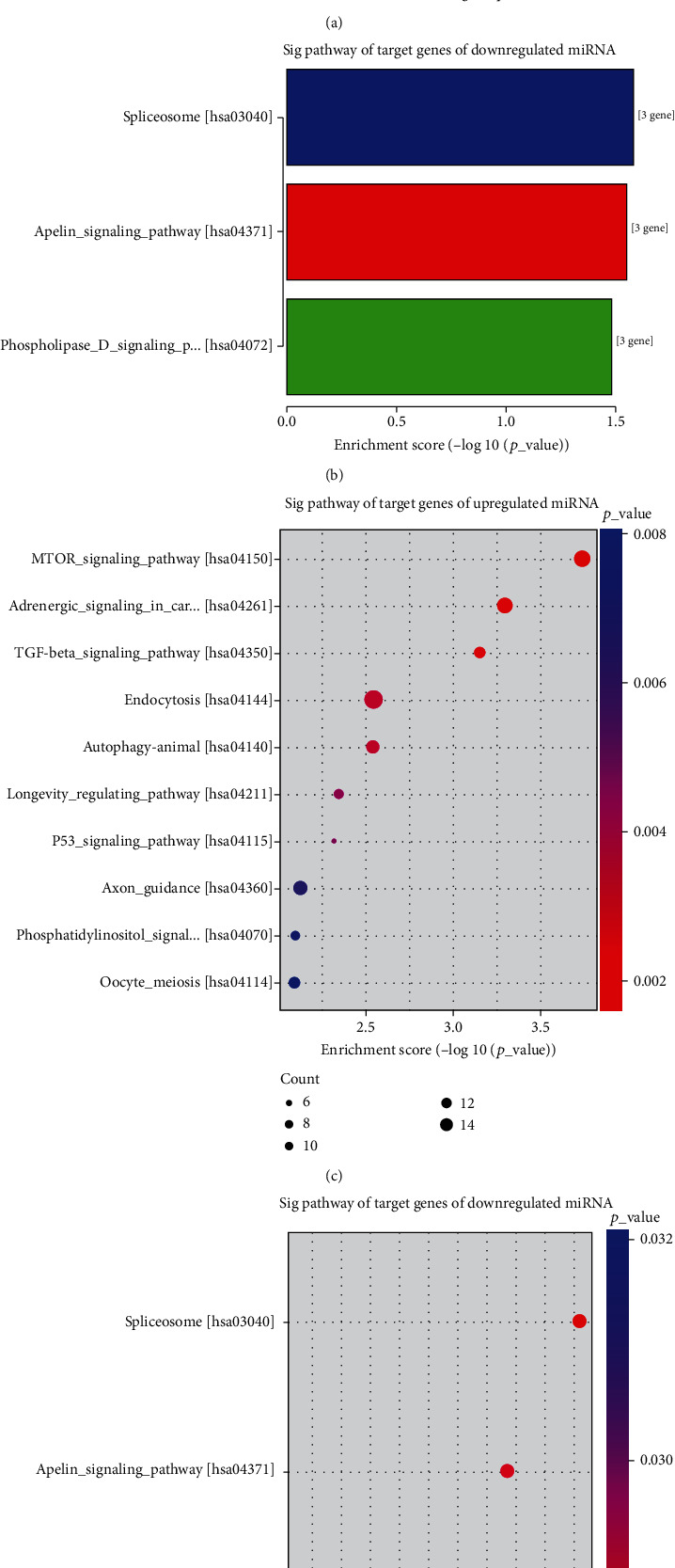
KEGG pathway significantly enriched in the predicted target genes of differentially expressed exosomal miRNAs in CH patients. (a, c) Enriched top 5 pathways of differentially upregulated miRNA target genes in CH patients compared with CS. (b, d) Enriched top 3 pathways of differentially downregulated miRNA target genes in CH patients compared with CS. Size and color of the bubble represented the amount of differentially expressed genes enriched in the pathway and enrichment significance, respectively.

KEGG enrichment analysis demonstrated that target genes were significantly enriched in 54 signaling pathways, of which “mTOR signaling_pathway” (12 genes, *P* = 1.83*E* − 04) was the most significantly enriched pathway of upregulated miRNA target genes (Figures 3(a) and 3(c), Table S3), and “spliceosome” (three genes, *P* = 2.62*E* − 02) was the most significantly enriched pathway of downregulated miRNA target genes (Figures 3(b) and 3(d), Table S3). These results suggest that CH has various genetic and phenotypic characteristics.

### 3.4. Real-Time qPCR Validation of Differentially Expressed Exosomal miRNAs

**Figure 4:**
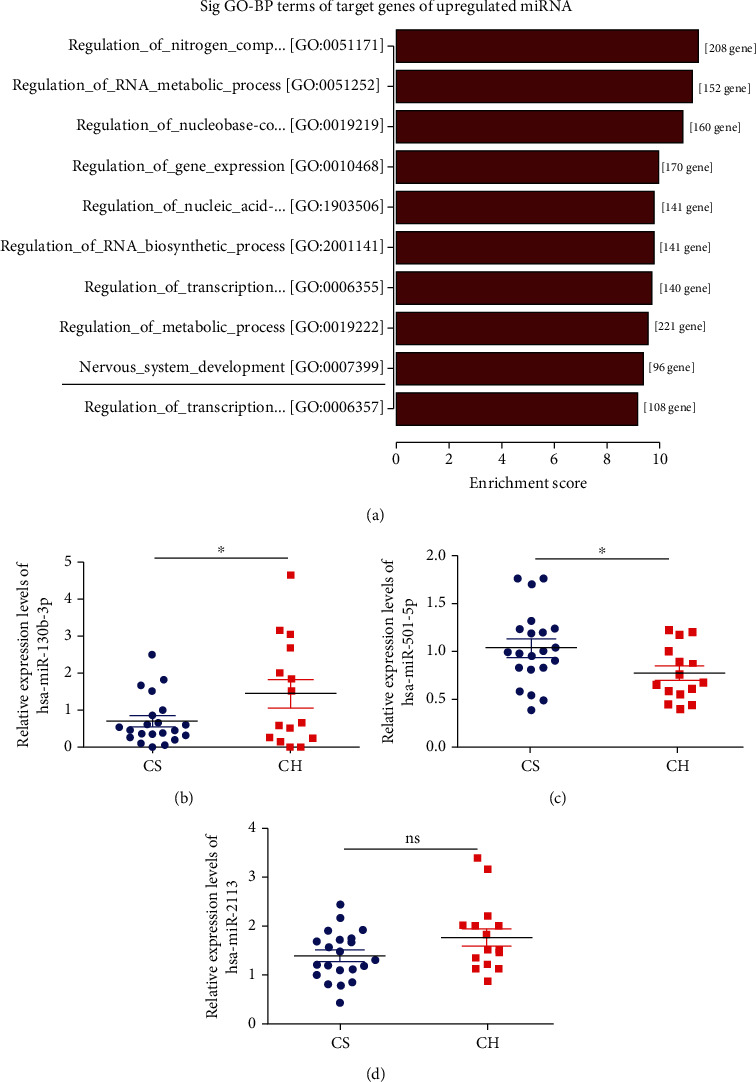
Real-time qPCR validation of differentially expressed exosomal miRNAs. (a) The GO-BP term “nervous system development” indicated by the black line was our concern. (b, c, d) The relative expression levels of hsa-miR-130b-3p, hsa-miR-501-5p, and hsa-miR-2113 in CH patients compared with CS by real-time qPCR. Values are represented as the mean ± SEM. *n* = 3; ^∗^*P* < 0.05.

Among these signaling pathways, we selected PTEN as a target gene in “nervous system development”, because this has been related to hydrocephalus in previous reports [17] (Figure 4(a), Figure S1). The miRNA corresponding to PTEN was hsa-miR-130b-3p. In addition, hsa-miR-2113 and hsa-miR-501-5p were noticeably differentially expressed in the CH patients (Table 4). Future studies should be carried out focusing on larger samples at an individual level. We performed real-time qPCR validation of these three miRNAs in cerebrospinal fluid exosomes from 15 CH patients and 21 CS and revealed that hsa-miR-130b-3p was upregulated, while hsa-miR-501-5p was downregulated, in CH patients compared with CS (Figures 4(b) and 4(c)). However, there was no significant difference in hsa-miR-2113 expression between CH patients and CS (Figure 4(d)). The trends in expression levels of these two miRNAs according to qPCR were in accordance with the miRNA sequencing results.

### 3.5. Upregulation of hsa-miR-130b-3p Decreased Expression of PTEN via the Predicted Binding Site

**Figure 5:**
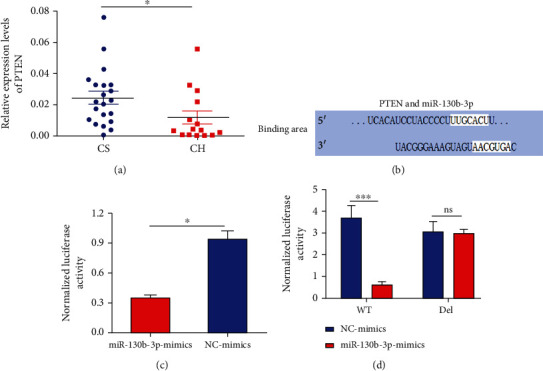
Upregulation of hsa-miR-130b-3p decreased expression of PTEN via the predicted binding site. (a) PTEN was downregulated in the 15 CH patients compared with the 21 CS by qPCR analysis. (b) The predicted binding site of PTEN and hsa-miR-130b-3p. The white area indicates the binding site of PTEN and hsa-miR-130b-3p. (c, d) The normalized luciferase activity of PTEN following transfection with hsa-miR-130b-3p mimics and NC mimics. Values are represented as the mean ± SEM. *n* = 3; ^∗^*P* < 0.05 and ^∗∗∗^*P* < 0.001.

PTEN is a potential target gene of hsa-miR-130b-3p. PTEN was downregulated in the 15 CH patients compared with 21 CS, according to the real-time qPCR results, in contrast to the trend for hsa-miR-130b-3p (Figure 5(a)). The TargetScan showed the predicted binding sites in the 3′-UTR of PTEN with hsa-miR-130b-3p (Figure 5(b)). hsa-miR-130b-3p mimics significantly suppressed luciferase reporter activity compared with NC mimics, after transfection with pGL3-promoter-PTEN-3′-UTR (Figure 5(c)). To avoid unspecific binding, the binding site in pGL3-promoter-PTEN-3′-UTR was deleted. Transfection of hsa-miR-130b-3p mimics significantly inhibited pGL3-promoter-PTEN-3′-UTR-WT activity, but had no effect on pGL3-promoter-PTEN-3′-UTR-Del activity (Figure 5(d)). These results show that upregulation of hsa-miR-130b-3p regulated downregulation of PTEN via the predicted binding site.

## 4. Discussion

Extracellular circulating miRNAs exist in most human body fluids, including cerebrospinal fluid, and are highly stable [18]. The delivery of miRNAs to recipient cells in circulating exosomes provides a novel method of intercellular communication. Dysregulation of exosomal miRNAs is an emerging element in a number of diseases, which reveals the important roles of exosomal miRNAs in both physiological and pathological pathways [9]. Several studies have implicated the function of exosomal miRNAs in some kinds of hydrocephalus. For example, Spaull et al. provided the first evidence for exosomes and exosomal miRNA expression in the cerebrospinal fluid in patients with posthemorrhagic hydrocephalus, and the increase in miR-1991-5P following the development of posthemorrhagic hydrocephalus made this an interesting potential biomarker [19]. hsa-miR-4274 was identified as a potential cerebrospinal fluid biomarker for idiopathic normal pressure hydrocephalus, with diagnostic potential, as well as the ability to predict the response to shunt treatment [20]. A prolonged elevation was shown in grade IV vs. grade III of intraventricular hemorrhage with higher miR-155 and miR-181b expression in cerebrospinal fluid at days 41-60 after intraventricular hemorrhage. These alterations may contribute to the development of later clinical complications in this clinical condition [21]. However, the role of exosomal miRNAs in the cerebrospinal fluid of patients with CH remains unclear. We therefore sequenced exosomal miRNAs in cerebrospinal fluid samples from three CH patients and three CS to compare the miRNA expression profiles and explore their functions in CH. As it has been reported that age and gender differences affect the expression pattern of miRNA in exosomes, the CH patients were compared with the age-sex-matched CS in our study [22, 23]. We identified 31 significantly expressed exosomal miRNAs in CH, including 26 that were upregulated and 5 that were downregulated. Among these differentially expressed miRNAs, hsa-miR-2113 and hsa-miR-501-5p were the most significantly upregulated and downregulated, respectively. Previous studies indicated that hsa-miR-2113 was associated with epithelial-mesenchymal transition in diabetes [24] and hepatocellular carcinoma [25], while hsa-miR-501-5p played an important role in modulating tumor progression, e.g., in hepatocellular carcinoma [26], gastric cancer [27], and colorectal cancer [28]. However, their roles in CH have not been investigated.

Because the main mechanism of miRNA is that it can recognize the target mRNA through base complementary pairing and guide the silencing complex to degrade the target mRNA or block the translation of the target mRNA according to the degree of complementarity, we predicted the target mRNAs of differentially expressed exosomal miRNAs and carried out GO and KEGG pathway enrichment analyses to reveal the biological processes and functions of the target mRNAs. Then, we found hsa-miR-130b-3p was predicted to be combined with *PTEN*, which was enriched in the “nervous system development” pathway. hsa-miR-130b-3p is known to be involved in cancer progression and various inflammatory diseases [29], and no studies have reported on the association between hsa-miR-130b-3p and CH; *PTEN* is located on 10q23.3 and encodes a lipid phosphatase with important roles in intracellular signal transduction through dephosphorylation of substrates such as Akt and S6 kinase [30]. Previous studies have suggested that PTEN was required for brain formation, and that dysregulation of PTEN resulted in abnormal brain development and progressive hydrocephalus [17]. A novel germline mutation of the PTEN gene is associated with VATER hydrocephalus syndrome [31]. And hsa-miR-130b-3p has been reported to negatively regulate PTEN by binding to the 3′-UTR in PTEN [29–31]. These suggested that hsa-miR-130b-3p was likely to play a pivotal role in the development of CH by targeting PTEN.

We therefore selected these three miRNAs (hsa-miR-2113, hsa-miR-501-5p, and hsa-miR-130b-3p) and PTEN for real-time qPCR validation. Because the cerebrospinal fluid samples were difficult to collect and susceptible to infection, we only collected 15 CH patients and 21 CS for real-time qPCR validation. hsa-miR-130b-3p and hsa-miR-501-5p were upregulated and downregulated, respectively, in CH patients compared with CS, in accordance with the miRNA sequencing results, while there was no significant difference for hsa-miR-2113. miRNA sequencing is a screening method in small samples, and the results of comparison between groups only suggest the possible differences and the false positive results also exist. Furthermore, real-time qPCR showed that PTEN was downregulated in CH patients, in contrast to the trend for hsa-miR-130b-3p. Dual-luciferase reporter assay showed that hsa-miR-130b-3p regulated the expression of PTEN by binding to the predicted site on the 3′-UTR. Therefore, upregulation of hsa-miR-130b-3p may be involved in the development of CH via interacting with PTEN and mediating its downregulation.

To the best of our knowledge, this study provides the first report of the expression profiles of exosomal miRNAs in CH. Exosomal hsa-miR-130b-3p and hsa-miR-501-5p may be involved in the development of CH. The mechanism of hsa-miR-130b-3p in CH has also been partly revealed. These findings will help to provide new diagnostic and therapeutic targets for CH.

## Figures and Tables

**Table 1:** Basic information of samples for miRNA sequencing.

Patient ID	Age	Sex	Samples	Phenotype
CH1	15 months	Female	15 ml cerebrospinal fluid	Congenital hydrocephalus
CH2	2 months	Male	15 ml cerebrospinal fluid	Congenital hydrocephalus
CH3	13 months	Male	15 ml cerebrospinal fluid	Congenital hydrocephalus
CS1	14 months	Male	15 ml cerebrospinal fluid	Intracranial space-occupying lesions
CS2	13 months	Female	13 ml cerebrospinal fluid	Tethered cord syndrome
CS3	10 months	Male	15 ml cerebrospinal fluid	Intracranial space-occupying lesions

CH: congenital hydrocephalus; CS: control subjects.

**Table 2:** Basic information of CH patients.

Patient ID	Age	Sex	Samples	Phenotype
CH1	15 months	Female	15 ml cerebrospinal fluid	Hydrocephalus
CH2	2 months	Female	15 ml cerebrospinal fluid	Hydrocephalus
CH3	14 months	Male	15 ml cerebrospinal fluid	Hydrocephalus
CH4	9 months	Female	7 ml cerebrospinal fluid	Hydrocephalus
CH5	29 days	Male	7 ml cerebrospinal fluid	Hydrocephalus
CH6	5 months	Male	15 ml cerebrospinal fluid	Hydrocephalus
CH7	6 months	Male	7 ml cerebrospinal fluid	Hydrocephalus
CH8	4 months	Female	8 ml cerebrospinal fluid	Hydrocephalus
CH9	24 months	Male	15 ml cerebrospinal fluid	Hydrocephalus
CH10	9 months	Female	8 ml cerebrospinal fluid	Hydrocephalus
CH11	24 months	Female	15 ml cerebrospinal fluid	Hydrocephalus
CH12	60 months	Male	25 ml cerebrospinal fluid	Hydrocephalus
CH13	15 months	Female	11 ml cerebrospinal fluid	Hydrocephalus
CH14	14 months	Male	9 ml cerebrospinal fluid	Hydrocephalus
CH15	2 months	Male	9 ml cerebrospinal fluid	Hydrocephalus

CH: congenital hydrocephalus.

**Table 3:** Basic information of CS.

Patient ID	Age	Sex	Samples	Phenotype
CS1	15 months	Male	15 ml cerebrospinal fluid	Intracranial space-occupying lesions
CS2	12 months	Female	13 ml cerebrospinal fluid	Congenital tethered cord syndrome
CS3	17 months	Male	15 ml cerebrospinal fluid	Intracranial space-occupying lesions
CS4	5 months	Male	8 ml cerebrospinal fluid	Congenital tethered cord syndrome
CS5	14 months	Male	15 ml cerebrospinal fluid	Intracranial space-occupying lesions
CS6	22 months	Female	15 ml cerebrospinal fluid	Congenital tethered cord syndrome
CS7	6 days	Male	15 ml cerebrospinal fluid	Intracranial hemorrhage
CS8	4 months	Male	10 ml cerebrospinal fluid	Congenital tethered cord syndrome
CS9	108 months	Female	10 ml cerebrospinal fluid	Intracranial hemorrhage
CS10	24 months	Male	20 ml cerebrospinal fluid	Congenital tethered cord syndrome
CS11	36 months	Male	8 ml cerebrospinal fluid	Intracranial space-occupying lesions
CS12	48 months	Male	7 ml cerebrospinal fluid	Congenital tethered cord syndrome
CS13	21 months	Female	20 ml cerebrospinal fluid	Intracranial hemorrhage
CS14	9 days	Male	5 ml cerebrospinal fluid	Congenital tethered cord syndrome
CS15	72 months	Female	12 ml cerebrospinal fluid	Congenital tethered cord syndrome
CS16	12 months	Female	10 ml cerebrospinal fluid	Intracranial hemorrhage
CS17	2 months	Male	6 ml cerebrospinal fluid	Congenital tethered cord syndrome
CS18	60 months	Female	10 ml cerebrospinal fluid	Congenital tethered cord syndrome
CS19	20 days	Male	20 ml cerebrospinal fluid	Congenital tethered cord syndrome
CS20	23 months	Female	15 ml cerebrospinal fluid	Intracranial space-occupying lesions
CS21	30 months	Female	6 ml cerebrospinal fluid	Intracranial space-occupying lesions

CS: control subjects.

**Table 4:** Upregulated and downregulated miRNAs in the volcano plot.

Upregulated miRNAs		Downregulated miRNAs	
miRNA ID	Log_2_FC	miRNA ID	Log_2_FC
hsa-miR-2113	7.910966974	hsa-miR-501-5p	-6.405186884
hsa-miR-302d-3p	7.163506356	hsa-let-7e-3p	-3.172160369
hsa-miR-137-5p	3.781667006	hsa-miR-29c-5p	-2.72261609
hsa-miR-320e	3.453737277	hsa-miR-223-5p	-1.647330356
hsa-miR-320c	3.379260772	hsa-miR-584-5p	-1.59751174
hsa-miR-320c	3.373467094		
hsa-miR-320b	3.223989228		
hsa-miR-129-5p	3.194153868		
hsa-miR-129-5p	3.194153868		c
hsa-miR-320b	3.143871008		
hsa-miR-130b-3p	3.037326563		
hsa-miR-4429	3.015750545		
hsa-miR-320d	3.008370049		
hsa-miR-320d	2.93745457		
hsa-miR-412-5p	2.780774639		
hsa-miR-296-3p	2.607140495		
hsa-miR-708-3p	2.537461519		
hsa-miR-320a-3p	2.480146691		
hsa-miR-1224-5p	2.413574833		
hsa-miR-134-5p	2.381827533		
hsa-miR-1298-5p	2.24115725		
hsa-miR-760	2.091967229		
hsa-miR-136-5p	2.0757691		
hsa-miR-181a-3p	2.051552079		
hsa-miR-193a-5p	1.813698773		
hsa-miR-7704	1.784297108		

FC: fold change.

## Data Availability

The datasets used and/or analyzed during the current study are available from the corresponding authors on reasonable request.

## Supplementary Materials

Supplementary 1 Figure S1: the target gene PTEN in the significant enriched KEGG pathway (mTOR signaling pathway).

Supplementary 2 Table S1: the mainly enriched pathways of target genes of upregulated miRNAs in GO analysis. Table S2: the mainly enriched pathway of target genes of downregulated miRNA in GO analysis. Table S3: the mainly enriched pathways of target genes of dysregulated miRNAs in KEGG analysis.
