# Supplementary figures and images for: Expression Profiles of Exosomal MicroRNAs Derived from Cerebrospinal Fluid in Patients with Congenital Hydrocephalus Determined by MicroRNA Sequencing
Source: Dis Markers. 2022 Mar 4;2022:5344508. doi: 10.1155/2022/5344508 (PMC8966745; doi:10.1155/2022/5344508)

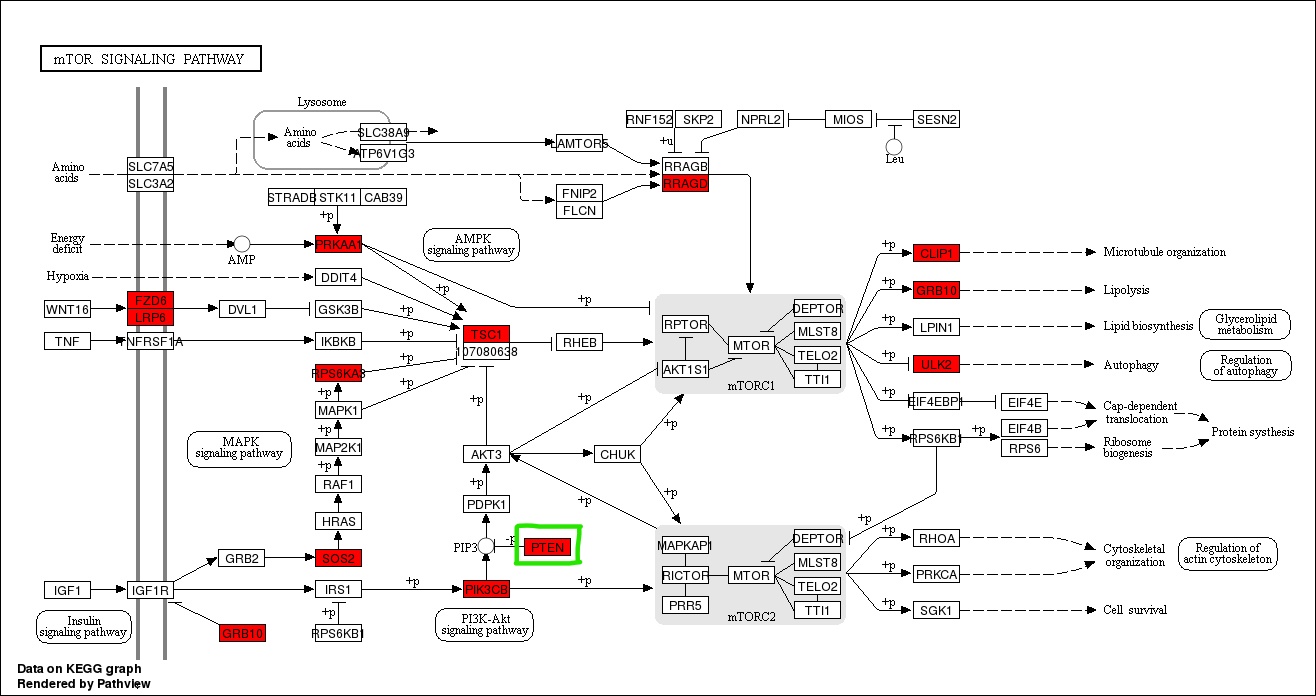

Supplement: Supplementary 1 — Figure S1: the target gene PTEN in the significant enriched KEGG pathway (mTOR signaling pathway). [file 5344508.f1.jpg]
