# Supplementary material for: Expression Profiles of Exosomal MicroRNAs Derived from Cerebrospinal Fluid in Patients with Congenital Hydrocephalus Determined by MicroRNA Sequencing
Source: Dis Markers. 2022 Mar 4;2022:5344508. doi: 10.1155/2022/5344508 (PMC8966745; doi:10.1155/2022/5344508)
Supplement: Supplementary 2 — Table S1: the mainly enriched pathways of target genes of upregulated miRNAs in GO analysis. Table S2: the mainly enriched pathway of target genes of downregulated miRNA in GO analysis. Table S3: the mainly enriched pathways of target genes of dysregulated miRNAs in KEGG analysis. [file 5344508.f2.docx]

| **Pathways** | **Gene counts** | **P value** |
| --- | --- | --- |
| Regulation of nitrogen compound metabolic process | 208 | 3.14E-12 |
| Regulation of RNA metabolic process | 152 | 5.21E-12 |
| Regulation of nucleobase-containing compound-metabolic process | 160 | 1.20E-11 |
| Nuclear lumen | 145 | 6.31E-10 |
| Nucleoplasm | 128 | 1.00E-09 |
| Intracellular organelle | 333 | 4.49E-08 |
| Double-stranded DNA binding | 47 | 4.34E-07 |
| RNA polymerase Ⅱ regulatory region sequence-specific DNA binding | 39 | 5.79E-07 |
| RNA polymerase Ⅱ regulatory region DNA binding | 39 | 7.13E-07 |

**Table. S1. The mainly enriched pathways of target genes of upregulated miRNAs in GO**

**Table. S2. The mainly enriched pathways of target genes of downregulated miRNAs in GO**

| **Pathways** | **Gene counts** | **P value** |
| --- | --- | --- |
| Response to water deprivation | 2 | 6.25E-04 |
| Tube morphogenesis | 10 | 8.12E-04 |
| Roof of mouth development | 4 | 8.13E-04 |
| Dendritic spine | 4 | 7.63E-03 |
| Neuron spine | 4 | 7.97E-03 |
| endosome | 10 | 1.29E-02 |
| HMG box domain binding | 2 | 3.18E-03 |
| Protein binding | 67 | 3.43E-03 |
| Peptide binding | 67 | 4.26E-03 |

**Table. S3. The mainly enriched pathways of target genes of dysregulated miRNAs in KEGG**

| **Pathways** | **Gene counts** | **P value** |
| --- | --- | --- |
| mTOR signaling pathway | 12 | 1.83E-04 |
| Adrenergic signaling in cardiomyocytes | 11 | 5.07E-04 |
| TGF-beta signaling pathway | 8 | 7.09E-04 |
| Endocytosis | 14 | 2.89E-03 |
| Autophagy-animal | 9 | 2.90E-03 |
| Spliceosome | 3 | 2.62E-02 |
| Apelin signaling pathway | 3 | 2.77E-02 |
| Phospholipase D signaling pathway | 3 | 3.27E-02 |
